# Impact of immune checkpoint inhibitors (ICIs) therapy on interferon-γ release assay (IGRA) and diagnostic value in non-small cell lung cancer (NSCLC) patients

**DOI:** 10.1186/s12890-024-02980-4

**Published:** 2024-04-12

**Authors:** Yijiao Xu, Qingwei Zhang, Zhisheng Chen, Shuwen Yang, Haiyan Chen, Xiong Xiao, Hongni Jiang

**Affiliations:** 1 Zhongshan Hospital (Xiamen), Fudan University, Xiamen, China; 2 Xiamen Clinical Research Center for Cancer Therapy, Xiamen, China; 3 Zhongshan Hospital, Fudan University, Shanghai, China; 4 Fudan Zhangjiang Institute, Shanghai, China

**Keywords:** Non-small cell lung cancer (NSCLC), Immune checkpoint inhibitors (ICIs), T-SPOT, Tuberculosis, Reactivation, Treatment outcomes

## Abstract

**Background:**

Tuberculosis (TB), a highly contagious respiratory disease, presents a significant global health threat, with a notable increase in incidence reported by the WHO in 2022. Particularly, the interplay between TB and non-small cell lung cancer (NSCLC) gains attention, especially considering the rising use of immune checkpoint inhibitors (ICIs) in cancer treatment. This interplay may influence TB diagnostics and reactivation, warranting a closer examination.

**Methods:**

A retrospective analysis was conducted on clinical data of NSCLC patients with positive T-SPOT results before undergoing anti-tumor treatment at Zhongshan Hospital (Xiamen), Fudan University, from January 1, 2021 to December 31, 2022. We assessed the incidence of tuberculosis reactivation and treatment outcomes among these patients. Moreover, we compared the differences in tuberculosis activity between the ICIs and non-ICIs treatment groups. Additionally, we observed the changes in T-SPOT spot count before and after immunotherapy, analyzing their association with tuberculosis activity and prognosis.

**Results:**

A total of 40 NSCLC patients with positive T-SPOT results before treatment were included in the study, with 26 patients in the ICIs treatment group and 14 patients in the non-ICIs treatment group. The study found no significant differences between the two groups in terms of gender, age, stage, histological type, performance status, driver gene expression, and distant metastasis. With a median follow-up time of 10.0 (6.0-14.5) months, three cases (11.5%) in the ICIs treatment group developed tuberculosis activity, diagnosed at 2, 3, and 12 months after ICIs treatment initiation. Conversely, no tuberculosis activity was observed in the non-ICIs treatment group, and the difference between the two groups was not significant (*P* = 0.186). Among the 32 patients who received ICIs treatment, spot count dynamics were diverse: four cases (12.5%) showed an increase, 12 cases (37.5%) had no change, and 16 cases (50.0%) had a decrease. During the follow-up, the progression rate (PD) was 50.0%, 75.0%, and 62.5% in the three groups, respectively (*P* = 0.527). Similarly, the mortality rate was 0%, 25.0%, and 25.0%, respectively (*P* = 0.106). Interestingly, among the patients with decreased spot counts, three cases (18.75%) were diagnosed with active pulmonary tuberculosis.

**Conclusions:**

For NSCLC patients with a positive T-SPOT response undergoing ICIs treatment, our study observed indications of active tuberculosis. The varied T-SPOT spot count changes post-ICIs treatment suggest a complex interaction, potentially linking T-SPOT spot count reduction to tuberculosis reactivation risk. These preliminary findings underscore the importance of further research to more accurately assess T-SPOT’s diagnostic utility in this context.

## Background

Tuberculosis (TB) is a highly contagious respiratory disease that presents a grave global threat. The 2022 report from the World Health Organization (WHO) reveals an unsettling change in the TB incidence trend, which had been declining over the past two decades. Contrary to this previous trend, there was an alarming increase of 3.6% in TB incidence from 2020 to 2021, affecting approximately 10.6 million individuals worldwide [1]. Research has shown that individuals with a history of TB are at a significantly higher risk of developing lung cancer. In fact, the incidence of lung cancer is increased by 3–10 times in patients with a previous TB infection compared to those without [2, 3]. In lung cancer patients, TB can manifest either as a concurrent or a latent infection, complicating the treatment of the tumor [4, 5].The Interferon-Gamma Release Assay (IGRA) is one of the diagnostic methods used for detecting tuberculosis infection and is recommended by the WHO as a primary in vitro immunological test for diagnosing Mycobacterium tuberculosis(MTB, commonly known as TB) infection, with T-SPOT.TB being one of the most commonly used versions. This assay functions by detecting the production of IFN-γ by effector T cells in the peripheral blood of the subject following stimulation with MTB-specific antigens. The enzyme-linked immune spot assay (ELISPOT) is used to quantify the number of IFN-γ-releasing effector T cells, which determines the test result [6].

In recent years, immune checkpoint inhibitors (ICIs) have been increasingly used in the treatment of various malignancies, including lung cancer and melanoma [7, 8]. ICIs have the potential to release the suppression of T cells in the human body, enhancing the anti-tumor activity of the immune cells. This mechanism could also enhance the anti-mycobacterial activity against tuberculosis. However, previous literature has shown an increased incidence of active TB or conversion to positive results in interferon-gamma release assays (IGRAs) during ICIs treatment [9]. The prolonged use of ICIs in NSCLC patients can lead to T cell activation and exhaustion, reducing T cell numbers and potentially increasing susceptibility to T cell-dependent infectious diseases [10, 11]. Given that T-SPOT results are also dependent on T cells. Thus, ICIs therapy in lung cancer patients could potentially affect T-SPOT outcomes, thereby impacting the diagnosis of pulmonary tuberculosis.

To date, the relationship between ICIs therapy in lung cancer and T-SPOT outcomes remains underexplored. A solitary prospective study reported a 3.3% incidence of T-SPOT conversion (from negative to positive) in lung cancer patients undergoing ICIs therapy, with 1.6% developing active pulmonary tuberculosis. However, this study included only 18 cases with positive T-SPOT results before treatment [12] without delving into the relationship between T-SPOT outcomes, immunotherapy efficacy, and prognosis.

Consequently, this research focuses on a T-SPOT.TB study aiming to monitor T-SPOT before and after ICIs therapy to examine the impact of ICIs treatment on the outcomes of the interferon-gamma release assay and its diagnostic efficacy in NSCLC patients.

## Methods

### Study design and study approval

The medical records of patients with non-small cell lung cancer (NSCLC) who received treatment at Zhongshan Hospital (Xiamen) Fudan University between January 1, 2021 and December 31, 2022 were retrospectively reviewed. Data were collected from our electronic medical record and patient follow-up. The data collected for all patients included age, sex, performance status (PS), tumor-node-metastasis (TNM) staging, histology, treatment regimens, survival, and adverse events.

Inclusion Criteria:1) Age greater than 18 years. 2) Diagnosis of stage IV NSCLC or stage IIIB/IIIC NSCLC that was either unresectable or had postoperative recurrence, confirmed both pathologically and radiologically. 3) Completion of a minimum of 2 cycles of anti-tumor treatment, which could include immune checkpoint inhibitors (ICIs), chemotherapy, or combination therapy. 4) Completion of T-SPOT testing prior to the commencement of treatment. 5) Availability of complete and standardized treatment and follow-up data. Exclusion Criteria:1) Diagnosis of small cell lung cancer (SCLC). 2) Presence of severe comorbidities affecting vital organs such as the heart, brain, liver, or kidneys. 3) Diagnosis with active pulmonary tuberculosis or extrapulmonary tuberculosis prior to treatment. 4) Required long-term (defined as continuous use for more than 4 weeks) oral or intravenous administration of corticosteroids. 5) Current treatment with other immunosuppressive agents.

This study was approved by the ethics committee of Zhongshan Hospital, Xiamen Branch, Fudan University (No. B2022-068).

### Evaluation of responses

We evaluated the overall survival (OS) and progression-free survival (PFS) among patients. OS was defined as the duration from the commencement of immunotherapy until death from any cause. Similarly, PFS was determined as the interval from the initial dose of immunotherapy to either the documented progression of the disease or death, whichever occurred first. The assessment of treatment efficacy was conducted according to the Response Evaluation Criteria in Solid Tumors (RECIST) version 1.1 [13]. Data analysis was conducted up to the censored date of May 31, 2023, with a median follow-up duration of 13.0 (interquartile range: 9.5–18.5) months.

### Assessment of T-SPOT.TB

In this study, the T-SPOT.TB assay (Oxford Immunotec Ltd., UK) was utilized. Initially, fresh venous blood samples were collected from the patients, and peripheral blood mononuclear cells (PBMCs) were isolated. These cells were then resuspended in AIM-V medium to achieve a concentration of 2.5 × 10^5 cells/mL. For the assay, 50 µL of this cell suspension was dispensed into each of the four wells of an IFN-γ antibody-coated microplate. AIM-V medium acted as the negative control, while PHA served as the positive control. ESAT-6 and CFP-10 were added to test wells A and B, respectively. The microplate was then incubated at 37 °C in a CO2 incubator for 16–20 h. After incubation, the microplate was washed, and an alkaline phosphatase-conjugated secondary antibody was applied. Subsequently, the plate underwent another incubation at 4 °C for 1 h and was washed with PBS. To visualize the results, BCIP/NBT substrate solution was added, inducing a color change. The spots formed were counted and interpreted using a microscope. A test was considered positive if: (1) the net number of spots in the test well (after subtracting the count from the negative control well) was ≥ 6, provided the count in the negative control well was between 0 and 5, or (2) the count in the test well was at least double that in the negative control well, assuming the negative control had ≥ 6 spots.

### Acid-fast staining of sputum smears

Sputum specimens were collected from patients in the early morning, with a focus on collecting purulent sputum. When required, specimens were diluted with normal saline and preserved in a sterile, sealed container. For analysis, a small aliquot of the specimen was carefully placed on a glass slide and subjected to the acid-fast staining technique. Following staining, the slide was meticulously examined under a microscope, with 100 fields of view being scrutinized. A result was deemed positive if 3 or more acid-fast bacilli were identified within any single field of view.

### Statistical analysis

For the analysis of continuous data that followed a normal distribution, the mean and standard deviation were represented as (X ± SD). In contrast, for continuous data not adhering to a normal distribution, the median and interquartile range (IQR) served as the descriptive statistics. Categorical data were summarized using frequencies and percentages. To compare continuous data across groups, t-tests or non-parametric tests were employed based on the data distribution. For categorical data comparisons, the chi-square test was the primary method used. In instances where the expected frequency in any table cell was below 5, Fisher’s exact test was utilized instead. A p-value of less than 0.05 was deemed to indicate statistical significance. All statistical analyses were conducted using SPSS software, version 26.0.

## Results

### Impact of ICIs therapy on tuberculosis activity in T-SPOT-positive NSCLC patients

#### Patient characteristics

**Fig. 1:**
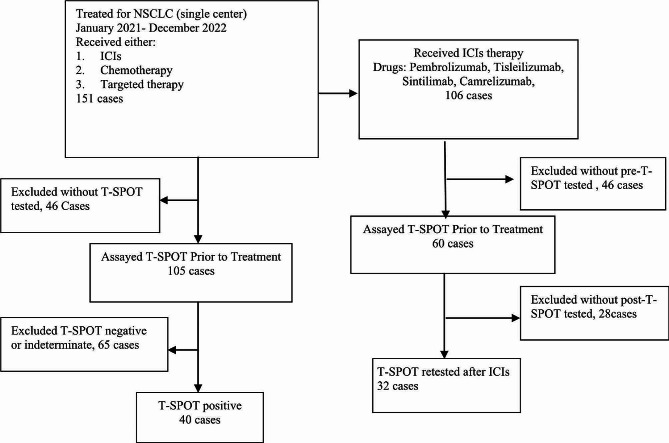
Flow chart showing cohort selection

In this study, a cohort of 40 T-SPOT-positive non-small cell lung cancer (NSCLC) patients was analyzed before the initiation of any treatment (Fig. 1). The gender distribution was predominantly male with 32 (80.0%) patients, while females accounted for 8 (20.0%). The median age of the participants was 65 years, with a division of 18 patients (45.0%) below 65 years and 22 patients (55.0%) aged 65 or above. According to the Eastern Cooperative Oncology Group Performance Status (ECOG-PS) scale, 16 patients (40.0%) were scored at 0, indicating fully active, able to carry on all pre-disease performance without restriction, whereas 24 patients (60.0%) had a score of 1 or higher, reflecting some limitations in physical activity. Histological analysis revealed that 23 patients (57.5%) had squamous cell carcinoma, 24 (60.0%) had adenocarcinoma, and the remaining 3 presented with other histological types. Staging information indicated that 11 patients (27.5%) were at stage III, while a larger group of 29 (72.5%) was at stage IV. Metastasis patterns showed that 8 patients (20.0%) had brain metastasis, 13 (32.5%) had bone metastasis, and 2 (5%) had liver metastasis. Additionally, 19 patients (47.5%) were found to have positive results for driver gene mutations. During the treatment course, targeted therapy was administered to 12 patients (30.0%). It was noted that none of the patients had been diagnosed with active pulmonary tuberculosis prior to treatment initiation.

For the purpose of this study, the patients were divided into two groups based on whether they received ICIs treatment or not: the ICIs treatment group and the non-ICIs treatment group. Comparative analysis between the two groups showed no statistically significant differences in terms of gender, age, stage of cancer, histological type, performance status score, presence of metastasis, or driver gene status (Table 1).

**Table 1:** Demographic and clinical characteristics of patients with advanced-stage lung cancer

Characteristics	All patients (*N* = 40)	ICIs Therapy (*n* = 26)	Non-ICIs Therapy (*n* = 14)	P value
Age, years				0.125
<65	18(45.0)	14	4	
≥ 65	22(55.0)	12	10	
Sex				0.068
Male	32(80.0)	23	9	
Female	8(20.0)	3	5	
ECOG-PS				0.685
0	16(40.0)	11	5	
≥ 1	24(60.0)	15	9	
Histopathology				0.179
Adenocarcinoma	24(60.0)	13	11	
Squamous cell carcinoma	13(32.5)	11	2	
other	3(7.5)	2	1	
TNM				0.170
Stage-III	11(27.5)	9	2	
Stage-IV	29(72.5)	17	12	
Metastasis				0.281
Brain	8(20.0)	5	3	
Bone	13(32.5)	7	6	
Liver	2(5.0)	0	2	
Driver genes Positive	19(47.5)	10	9	0.817

### Tuberculosis activity

Among the patients in the ICIs treatment group, a total of 3 cases (11.5%) were diagnosed with active pulmonary tuberculosis during follow-up, occurring at intervals of 2 months, 3 months, and 12 months after ICIs treatment initiation. In contrast, no cases of active pulmonary tuberculosis were observed in the non-ICIs treatment group. However, the difference between the two groups did not reach statistical significance (*P* = 0.186, Table 2).

**Table 2:** Tuberculosis activity between ICIs and Non-ICIs treatment group

	ICIs Therapy *n* = 26	Non-ICIs Therapy *n* = 14	P value
Active tuberculosis	3	0	0.186
Latent infection	23	14	

### Impact of ICIs treatment on T-SPOT spot

#### Patient characteristics

A total of 32 NSCLC patients were monitored for T-SPOT tests before and after immunotherapy (Fig. 1). Out of these, 30 (93.8%) were male, while 2 (6.2%) were female. The median age stood at 61 years, with a distribution where 19 patients (59.4%) aged below 65 years and 13 patients (40.6%) were aged 65 years or older. The ECOG-PS results showed an equal distribution, with 16 patients (50.0%) scoring 0 and another 16 patients (50.0%) scoring 1. In terms of histological analysis revealed that 15 patients (46.9%) had squamous cell carcinoma, 15 patients (46.9%) had adenocarcinoma, and 2 patients (6.2%) had adenosquamous carcinoma. As for the disease stage, 11 patients (34.4%) were classified as stage III, while 21 patients (65.6%) were classified as stage IV. The distribution of treatment agents was as follows: pembrolizumab (15.6%), tislelizumab (43.8%), camrelizumab (15.6%), and sintilimab (25.0%). Among the patients, 24 (75.0%) received first-line immunotherapy, while 8 (25.0%) received second-line treatment. Brain metastasis was observed in 7 patients (21.9%), bone metastasis in 9 patients (28.1%), and liver metastasis in 2 patients (6.2%). A total of 10 patients (31.2%) tested positive for driver gene mutations, and 3 patients (9.4%) had a history of prior targeted therapy. Importantly, none of the patients were diagnosed with active pulmonary tuberculosis prior to treatment (Table 3).

**Table 3:** Characteristics of 32 NSCLC patients with pre- and post-T-SPOT testing

Characteristics		*N* = 32	(%)
Age, years			
	<65	19	59.4
	≥ 65	13	40.6
Sex			
	Male	30	93.8
	Female	2	6.2
ECOG-PS			
	0	16	50.0
	1	16	50.0
Histopathology			
	Adenocarcinoma	15	46.9
	Squamous cell carcinoma	15	46.9
	Other	2	6.2
TNM			
	Stage-III	11	34.4
	Stage-IV	21	65.6
ICIs	Pembrolizumab	5	15.6
	Tislelizumab	14	43.8
	Camrelizumab	5	15.6
	Sintilimab	8	25.0
ICIs lines	First line	24	75.0
	Second line	8	25.0
Metastasis			
	Barin	7	21.9
	Bone	9	28.1
	Liver	2	6.4
Driver genes	Positive	10	31.2
Targeted therapy		3	9.4

### Changes in T-SPOT and prognostic analysis in NSCLC patients received ICIs treatment

As of May 31, 2023, among the 32 patients, 4 cases showed an increase in spot counts after treatment. Out of these, 2 cases were classified as PD, while no deaths were reported. Additionally, 12 patients had no change in spot counts after treatment, all of whom had a spot count of 0 before treatment. Among this group, 8 patients experienced PD, and 3 patients died, all due to tumor-related causes. Furthermore, 16 patients exhibited a decrease in spot counts after treatment. Among the 8 patients who had spot counts greater than 0 before treatment, 10 experienced PD, and 4 patients died. Notably, one death was attributed to infection with the novel coronavirus, while the remaining deaths were tumor-related causes. The proportions of disease progression in the three groups were 50.0%, 75%, and 62.5%, respectively, with a p-value of 0.837. The proportions of deaths were 0%, 25%, and 25% in the three groups, respectively, with a p-value of 0.527(Table 4).

**Table 4:** Changes in T-SPOT spot counts and prognosis in NSCLC patients undergoing ICIs treatment

T-SPOT Counts	Increase *n* = 4	No change *n* = 12	Decrease *n* = 16	*P* value
Prognosis, n(%)				
PD	2(50.0)	8(66.7)	10(62.5)	0.527
DCR	2(50.0)	4(33.3)	6(37.5)	
Dead	0(0.0)	3(25.0)	4(25.0)	0.837
Survival	4(100.0)	9(75.0)	12(75.0)	

### TB activity and T-SPOT results changes after ICIs treatment

During the follow-up period until the cutoff date, 3 cases (9.38%) of active pulmonary tuberculosis were diagnosed among NSCLC patients undergoing immune checkpoint inhibitors (ICIs) treatment. Of these cases, 2 were confirmed positive through sputum tuberculosis smear examination, while 1 case showed detection of mycobacterium tuberculosis via sputum metagenomic next-generation sequencing (mNGS). The onset of active pulmonary tuberculosis was noted at 2 months, 3 months, and 12 months after the initiation of ICIs treatment. Among the 16 patients with a decrease in T-SPOT spot count, 3 cases (18.75%) were diagnosed with active pulmonary tuberculosis. In detail, one case had a decrease in T-SPOT spot count from 16 to 0, another case had a decrease from 25 to 4, and the third case had a decrease from 44 to 18. Following diagnosis, all 3 patients temporarily discontinued immune therapy and initiated anti-tuberculosis treatment.

## Discussion

A comprehensive meta-analysis has indicated an elevated risk of tuberculosis in cancer patients when compared to the general population. Particularly in the realm of solid tumors, lung cancer stands out with a sixfold increased risk in patients [14]. Both lung cancer and tuberculosis share similar clinical manifestations, and there are cases where they can be diagnosed simultaneously or even be causally linked. Chronic inflammation, genomic changes, and fibrosis associated with tuberculosis are recognized as significant contributors to potential carcinogenic processes. Conversely, the progression of lung cancer and antitumor therapies may also trigger the reactivation of latent tuberculosis infections [15]. Moreover, the presence of untreated inactive tuberculosis lesions in the lungs, coupled with risk factors such as diabetes, smoking, and corticosteroid use, increases the risk for the reactivation of latent tuberculosis [16]. In a study conducted in Japan, among 39 lung cancer patients who subsequently developed tuberculosis (diagnosed more than one month after lung cancer), 37 presented risk factors for tuberculosis or previous tuberculosis infections, with 19 exhibiting two or more such factors [5]. Additionally, the combined use of anticancer drugs with other treatments [17] and the use of ICIs [18] have been linked to the risk of developing tuberculosis.

Previous evidence has suggested a potential association between ICIs treatment and an increased risk of developing pulmonary tuberculosis [19]. In our research, we detected 3 cases (9.38%) of active pulmonary tuberculosis during ICIs treatment. Interestingly, these patients showed a significant decrease in T-SPOT spot counts after ICIs therapy. Yet, the number of spots produced by Phytohemagglutinin (PHA), a non-specific stimulant that induces INF-γ release from T cells as a positive control, consistently remained above 100. This indicates a depletion of antigen-specific effector T lymphocytes following ICIs treatment, exposing patients to a dual risk of infection and tumor progression. Notably, one of the 3 cases of active pulmonary tuberculosis in our study succumbed to a severe SARS-CoV-2 infection. International studies have also highlighted an increased prevalence of infectious diseases reliant on T cell-mediated immunity in individuals with reduced T cell counts [10, 19].

Based on the IGRA results before treatment, tuberculosis disease activity can be divided into two categories: newly acquired and reactivation. In a retrospective study, approximately 1.7% (5/297) of lung cancer patients undergoing ICIs treatment developed active pulmonary tuberculosis during the course of therapy. Among these cases, 60% (3/5) were already T-SPOT positive even before initiating ICIs treatment [9]. This observation aligns with similar findings reported in another prospective cohort study, which posits ICIs treatment as a risk factor for the development and exacerbation of tuberculosis [12]. In our study, 3 cases of active tuberculosis were observed, and all had a positive T-SPOT result before treatment initiation, pointing to reactivation of latent tuberculosis infection (LTBI).

The occurrence of active tuberculosis following ICIs treatment may be linked not solely to ICIs-induced T cell depletion but also to the synergistic effects of various factors [20]. For example, the use of corticosteroids during treatment to manage immune-related adverse events may also independently heighten the risk of LTBI reactivation and progression. Furthermore, the prevalence of tuberculosis in a given region can affect the incidence of pulmonary tuberculosis following ICIs treatment. In exploring the discussion of immunological mechanisms, studies have demonstrated that mice lacking the PD-1 receptor are more vulnerable to Mycobacterium tuberculosis [21]. Contrary to the previously held notion that immune dysfunction leads to tuberculosis activation, the disruption of immune tolerance and excessive immune activation can equally play a role in the activation of Mycobacterium tuberculosis [22, 23]. The interaction between PD-1 and PD-L1 creates a state of immune tolerance within the body [24], yet, the use of PD-1 inhibitors in ICIs treatment disrupts this delicate balance. While it stimulates the body’s immune response against cancer cells, thus having an anticancer effect [25]. It may also lead to excessive inflammation and the release of inflammatory cytokines such as TNF-α, IL-18, and IFN-γ, which are implicated in tissue damage and cavity formation. These dynamics can further promote the occurrence and progression of tuberculosis [26].

The T-SPOT spot count in patients after ICIs treatment is determined by both the immune status of T lymphocytes and the burden of Mycobacterium tuberculosis within the body. As ICIs alleviate the inhibitory state of T lymphocytes and enhance their function and quantity, a persistent positive T-SPOT result or a conversion from negative to positive can be critical indicators of tuberculosis activation or reactivation [19]. Our study indicates that when T-SPOT shifts from positive to negative or when there is a decrease in spot count following ICIs treatment, patients may be experiencing antigen-specific activation and depletion of T lymphocytes. Hence, it is imperative to maintain high vigilance for the possibility of tuberculosis activation or reactivation. Routine screening and treatment for latent Mycobacterium tuberculosis infection ought to be conducted before and after ICIs treatment [27].

The implications of ICIs treatment on T-SPOT results remains uncertain, and prudence is advised when interpreting T-SPOT results in patients undergoing ICIs treatment, particularly in cases of T-SPOT conversion to negative or a decrease in spot count. Such changes do not necessarily signify an improvement in tuberculosis infection but may rather serve as a warning sign for tuberculosis activation.

Limitations of this study include its retrospective design and small sample size, which underscore the need for larger-scale, multicenter, or prospective studies to more comprehensively explore this topic. To broaden the research horizon, future studies can extend beyond NSCLC patients receiving ICIs treatment to include a wider range of tumor types, aiming to mitigate potential adverse effects of tuberculosis activation in cancer patients.

## Conclusions

In NSCLC patients with a positive T-SPOT response undergoing ICIs treatment, cases of active tuberculosis were observed. Post-ICIs treatment, the changes in T-SPOT spot count demonstrated variability, with a notably higher occurrence of active tuberculosis in patients who experienced a decrease in T-SPOT spot count. This highlights the need to re-evaluate the predictive value of T-SPOT for tuberculosis activity in NSCLC patients treated with ICIs through more extensive research.

## Data Availability

The datasets generated during and/or analyzed during the current study are available from the corresponding author on reasonable request.
